# Influence of Freedom of Movement on the Health of People With Dementia: A Systematic Review

**DOI:** 10.1093/geront/gnac114

**Published:** 2022-08-05

**Authors:** Suzan van Liempd, Marjolein Verbiest, Annerieke Stoop, Katrien Luijkx

**Affiliations:** Department of Tranzo Scientific Centre for Care and Wellbeing, Tilburg School of Social and Behavioral Sciences, Tilburg University, The Netherlands; Stichting Mijzo, Waalwijk, The Netherlands; Department of Tranzo Scientific Centre for Care and Wellbeing, Tilburg School of Social and Behavioral Sciences, Tilburg University, The Netherlands; Department of Tranzo Scientific Centre for Care and Wellbeing, Tilburg School of Social and Behavioral Sciences, Tilburg University, The Netherlands; Department of Tranzo Scientific Centre for Care and Wellbeing, Tilburg School of Social and Behavioral Sciences, Tilburg University, The Netherlands

**Keywords:** Liberty, Locked, Nursing home, Positive Health

## Abstract

**Background and Objectives:**

To protect residents with dementia from harm, nursing homes (NHs) often have closed-door policies. However, current research suggests a positive influence of freedom of movement, that is, the right to (decide to) independently move from one place to another, on the health of NH residents with dementia. This systematic review aims to collate, summarize, and synthesize the scientific evidence published to date on the influence of freedom of movement on health among NH residents with dementia.

**Research Design and Methods:**

Multiple databases were searched up until March 2021. Peer-reviewed qualitative, quantitative, and mixed methods studies were included. Health was operationalized using the Positive Health framework, encompassing 6 dimensions: bodily functions, mental functions and perception, existential dimension, quality of life, social and societal participation, and daily functioning. The quality of included studies was assessed using the Mixed Methods Appraisal Tool.

**Results:**

Sixteen studies were included of good to excellent quality. Compared to closed NHs, freedom of movement in semiopen and open NHs may have a positive influence on bodily functions, mental functions and perception, quality of life, and social and societal participation. The influence on daily functioning and on the existential dimension remains unclear.

**Discussion and Implications:**

Freedom of movement of NH residents with dementia is often studied as part of a larger context in which other factors may contribute to health benefits. More research is therefore needed to unravel the underlying mechanisms of the positive influence of freedom of movement on health.

## Background and Objectives

Around the globe, approximately 55 million people have dementia, and this number is expected to rise to 78 million in 2030 and 139 million in 2050 (World Health Organization, 2021). Persons with dementia have difficulty with several types of cognitive abilities, such as memory and orientation (Arvanitakis & Bennett, 2019). Poor cognition and behavioral and psychological symptoms of dementia (Toot et al., 2017), especially wandering (Cipriani et al., 2014), are the most significant reasons behind nursing home (NH) admissions. A NH is a facility with a domestic-style environment that provides 24-hr functional support and care for persons who require assistance with activities of daily living (ADL) and who often have complex health needs and increased vulnerability (Sanford et al., 2015). Despite this international definition of a NH, there are many differences around the world in terms of character of the home, values and regulations, staffing and character, and number of residents. For example, some NHs specialize in psychogeriatrics, while other NHs also accommodate residents with other chronic diseases, such as Parkinson’s and somatic disorders. Residents with dementia are often grouped together on a care unit, a geographic area in the NH with dedicated management (Estabrooks et al., 2011).

NHs range in the level of freedom of movement they provide for their residents with dementia. Literature often defines the level of movement related to open or closed doors (Calkins, 2018; Driessen et al., 2017). Most traditional NHs have closed doors motivated by preventing residents with dementia from getting lost and the higher risk of falls and accidents (Driessen et al., 2017; Evans et al., 2018; Landeweer et al., 2021; Wigg, 2010), even though research shows that in general a majority of NH residents spend at least part of their day in wheelchairs (Wick and Zanni, 2007) or lying down (Kennerly et al., 2022). These closed-door policies often do not consider the diversity of the NH resident population, in particular regarding characteristics that are associated with decisions about freedom of movements, such as the type and severity of dementia and physical ability to move. In closed settings, residents with dementia are free to move within a care unit but are not allowed to independently leave this unit without supervision (Steele et al., 2020). However, by keeping people with dementia safe behind closed doors, their freedom of movement, that is, the right to decide to independently move from one place to another, is restricted (Steele et al., 2020; Waal, 2014). Moreover, the literature suggests that being locked behind closed doors can have a negative influence on quality of life and autonomy of residents with dementia (Steele et al., 2020; Wigg, 2010). A previous scoping review suggests that limited freedom of movement is a frequently noted source of frustration among NH residents with dementia (Shiells et al., 2019). Consequently, residents may demonstrate resistance or distress; behaviors that are often labeled as “challenging” or as “behavioral and psychological symptoms of dementia” and that may in turn reinforce keeping them behind closed doors (Steele et al., 2020).

NHs that define wandering as part of person-centered care, embracing personal choice and autonomy, give residents with dementia free access to living spaces outside the care unit (Graham, 2017; Wigg, 2010). Surveillance technology such as location-tracking devices can be used to enhance this freedom of movement in this open-door setting (Niemeijer et al., 2014; Wigg, 2020). However, an open-door setting still can have closed doors, for instance, when residents are allowed to move independently within the NH building and/or enclosed garden but are not allowed to enter the outside world. In this semiopen setting, residents have free access to outdoor spaces within the NH environment, such as gardens and outdoor covered and uncovered areas (Van den Berg et al., 2020). In an open setting, residents are free to go wherever they like, including outside the NH environment; there are no closed doors (Driessen et al., 2017).

Dilemmas concerning autonomy and safety for NH residents with dementia continue to be a topic in NHs, in which the call to give residents more freedom of movement is growing (Aros, 2018; Graham, 2021; Kaldy, 2018; Landeweer et al., 2021). To support NHs in opening the doors, it is important to know if and how this will affect the health of people with dementia. According to the concept of Positive Health, health is defined as the ability to adapt and to self-manage in the face of social, physical, and emotional challenges (Huber et al., 2011), encompassing six dimensions: bodily functions, mental functions and perception, existential dimension, quality of life, social and societal participation, and ADL (Huber et al., 2016).

To date, little international scientific research exists specifically aiming to examine the influence of freedom of movement across this broad concept of the health of people with dementia in NHs. The few studies that have been published focus on only one or a few dimensions of health (i.e., bodily functions, mental functions, and perception). Therefore, the purpose of this systematic literature review is to provide an overview of what is known from the international scientific literature about the influence of freedom of movement on all health dimensions among people with dementia living within NHs.

## Research Design and Methods

### Search Strategy

To provide insights into current knowledge on the influence of freedom of movement on the health of people with dementia living in NHs, a systematic literature review was carried out. The research question was constructed by using the PICO strategy: (P) people with dementia living in NHs; (I) freedom of movement in semiopen and open settings; (C) freedom of movement in closed settings; and (O) health, defined as Positive Health, which encompasses six dimensions: bodily functions, mental functions and perception, existential dimension, quality of life, social and societal participation and daily functioning (Huber et al., 2016).

To structure the search and selection process, the Preferred, Reporting items of Systematic Reviews and Meta-Analyses (PRISMA) were followed (Moher et al., 2010). The search strategy was developed in embase.com, optimizing the search by comparing articles retrieved by thesaurus terms to those retrieved by the terms from the title and or abstract, identifying potentially relevant terms (Bramer, de Jonge et al., 2018; Bramer, Rethlefsen et al., 2018). Papers of interest were expected to have been published in biomedical, psychological, and health care management journals, so the following databases were searched from inception until March 2021: Medline, Embase, PsycINFO, Web of Science and Cochrane. The search terms were selected based on three categories: population (people with dementia), topic (influence of freedom of movement on health), and care setting (NH). A search string of synonyms and free text words based on these three categories was developed and programmed for each database (Online Supplementary Material).

### Eligibility Criteria

Studies regarding the influence of freedom of movement on the health of people with dementia in closed, semiopen, or open NHs, as operationalized in the introduction, using qualitative, quantitative, or mixed methods designs that were peer-reviewed and written in English were eligible for inclusion. Excluded from this review were studies in which the target group resides somewhere other than an NH, such as at home, daycare or in a hospital, studies that described restrictions of movement other than locked doors, such as belts in bed and/or in a wheelchair (Hofmann & Hahn, 2014), other systematic reviews and statements or were written in a language other than English. Also excluded were studies that examined freedom of movement across various groups where it is not clear which results apply specifically to residents with dementia.

### Study Selection and Data Extraction

In the first selection phase, duplicates were removed, and all titles and abstracts were screened by one reviewer (S. van Liempd). If all eligibility criteria were met or in any cases of doubt, articles proceeded for further screening. In the second screening phase, two reviewers (S. van Liempd and A. Stoop) assessed the abstracts of the publications independently using the eligibility criteria. Subsequently, two reviewers (S. van Liempd and A. Stoop) independently applied the inclusion criteria to all full-text articles that remained after the previous screening. In all steps of the study selection process, disagreements between the two reviewers were discussed until a consensus was reached. If no agreement between the two reviewers was reached, a third reviewer was consulted (M. Verbiest).

A format was agreed upon outlining the study aim, design, populations, level of freedom (closed, semiopen, and open setting), and the dimensions of Positive Health. With this format, data from the included articles were independently extracted by pairs of two reviewers per article (S. van Liempd and A. Stoop or M. Verbiest). If consensus between the two reviewers regarding extraction was not reached, a third reviewer was consulted (A. Stoop or M. Verbiest). Furthermore, we contacted one author for further information.

### Quality Assessment

To assess the methodological quality of the included studies, the Mixed Methods Appraisal Tool Checklist (MMAT; Hong et al., 2018) was used. The advantage of this instrument is that it allows an assessment of qualitative, quantitative, and mixed methods studies. The MMAT consists of two general screening questions and five specific methodological criteria for each type of research design. The included articles were assessed independently by pairs of two reviewers independently (S. van Liempd and A. Stoop or M. Verbiest). In cases of disagreement, a consensus was reached in discussions between the reviewers.

### Analysis

To analyze the impact of freedom of movement on the health of NH residents with dementia, first, each health outcome described in the included studies was categorized into one of the six dimensions of Positive Health (Huber et al., 2016). When a health outcome did not correspond to one of the listed aspects from Huber’s model, this health outcome was categorized under the best-fit dimension, based on consensus among the reviewers (S. van Liempd, M. Verbiest, or A. Stoop; Table 1). Second, the level of freedom of movement of the NH that was examined in each included study was divided into closed, semiopen, and open settings. Third, for each health dimension, health outcomes were related to the level of freedom of movement it applied to.

**Table 1.** Types of Health Outcomes Used in the Included Studies, Clustered into the Six Dimensions of Positive Health

Bodily functions	Mental functions and perception	Existential dimension	Quality of life	Social and societal participation	Daily functioning
Medical facts Medical observations Physical functioning Complaints and pain Energy **Physical activity** **Sleep** **Amount and severity of falls** **Physical harm**	Cognitive functioning: **Memory, language abilities, spatial abilities, orientation in time and place**	Meaning/meaningfulness Striving for aims/ideals Future prospects Acceptance	***Quality of life/well-being*** Experiencing happiness Enjoyment Perceived health Flourishing Zest for life Balance **Feeling safe and secure** **Feeling of captivity** **Sense of ownership** **Dignity** **Feeling dignified**	Social and communicative skills	Basic ***ADL*** Instrumental ADL Ability to work Health literacy
	Emotional state: **Anger, aggression, anxiety, agitation, mood, panic, frustration, disappointment, hysteria, affect**			Meaningful ***relationships***	
	Experiencing being in charge/manageability Self-management **Autonomy, being controlled** Resilience, sense of coherence **Depression** **Withdrawal** **Conflict** **Distress**			***Social contacts*** **Social interactions**	
				Experiencing being accepted	
				Community involvement	
				Meaningful work	
				**Meaningful activities**	

*Notes*: ADL = activities of daily living. Bolded aspects are aspects found in the included articles; non bolded aspects are aspects formulated by Huber et al. (2011). Italic and bolded aspects formulated by Huber et al. (2011) and found in the included articles.

## Results

### General Findings

**Figure 1.**
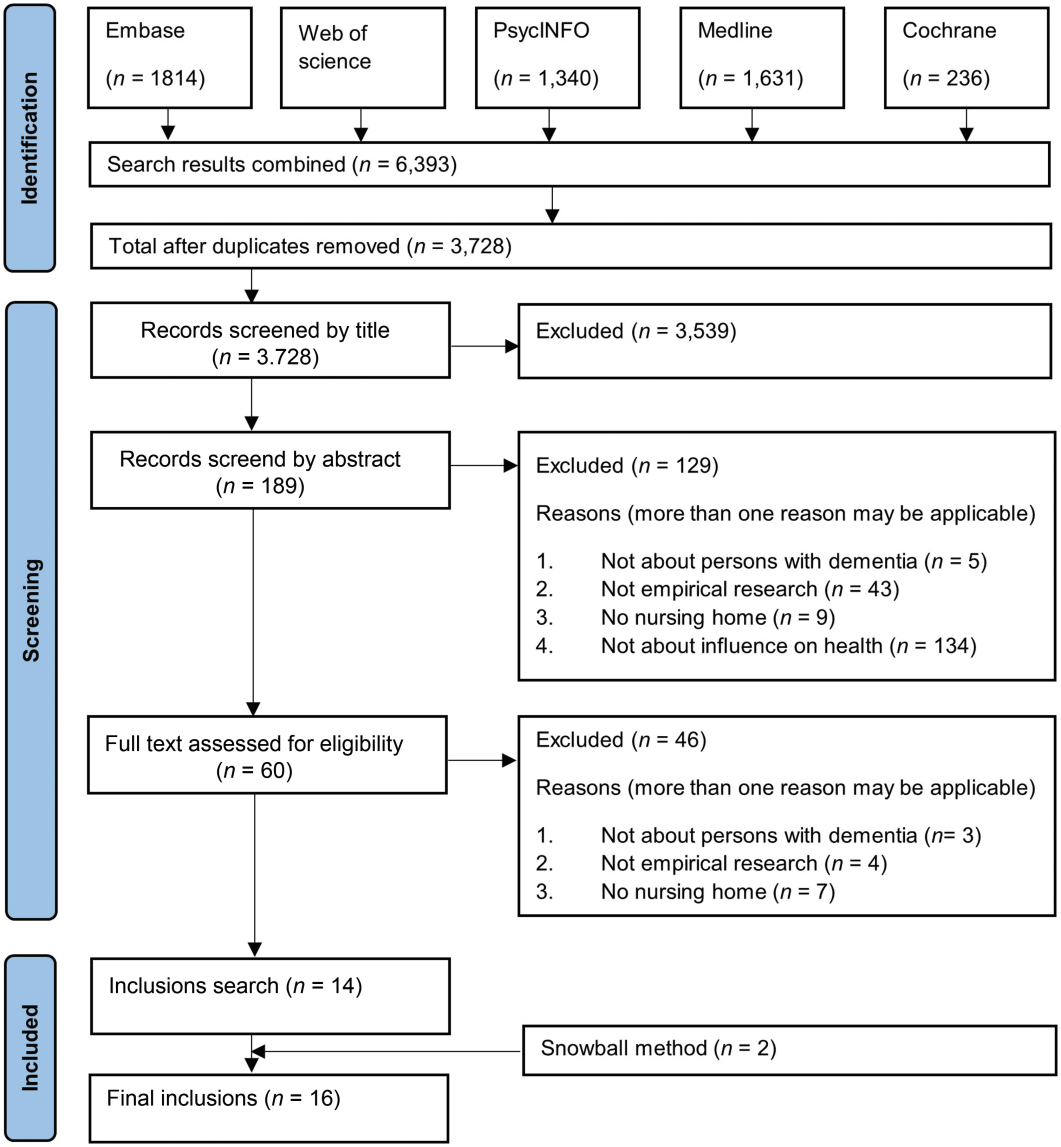
PRISMA flow diagram.

The search yielded 3,728 nonduplicate articles. Of these, 16 articles fit the inclusion criteria (Figure 1). Characteristics of the included studies are described in Table 2. Overall, the methodological quality of the included studies was good to excellent; four studies had a score of four out of five according to the MMAT, whilst all others fully met the MMAT criteria (Hong et al., 2018). As such, all studies were included in the analyses. The studies included were published between 2008 and 2020 in seven different countries; the majority were conducted in Europe (*n* = 9), followed by the United States of America (*n* = 6) and Australia (*n* = 2). Most studies described health outcomes on one or two dimensions of Positive Health (*n* = 11), and six studies described outcomes on three or four dimensions; there is no study that describes outcomes on five or six dimensions. Mental functions and perception are the most frequently examined (*n* = 12).

**Table 2.** Characteristics of Included Studies

Study; country	Study aim	Data collection method(s)	Sample (study population[s], [*n*])	Age, gender, level of dementia of residents	Dimensions of Positive Health described	MMAT^a^
Quantitative studies						
De Boer, Hamers, Zwakhalen, Tan, Beerens et al. (2017); The Netherlands	To investigate whether residents at green care farms participate more in (physical) activities and social interactions compared with residents of regular small-scale living facilities and traditional NHs.	Observations	Residents (at baseline *n* = 115; follow up *n* = 100)	Greencare farm: age not specified, 23 women and 11 men, moderate dementia Traditional NH: age not specified, 18 women and 11 men, moderate dementia Regular small-scale living facility: 45 women and seven men, moderate dementia	BF SSP	5
De Boer, Hamers, Zwakhalen, Tan et al. (2017); The Netherlands	To compare quality of care, quality of life and related outcomes in green care farms, regular small-scale living facilities and traditional NHs for people with dementia.	Medication and fall records; structure indicators; surveys	Residents (*n* = 115)	Greencare farm: age not specified, 23 women and 11 men, moderate dementia Traditional NH: age not specified, 18 women and 11 men, moderate dementia Regular small-scale living facility: 45 women and seven men, moderate dementia	MFP QoL SSP	5
Detweiler et al. (2008); USA	To explore the effect of adding a wander garden to an existing dementia unit on inappropriate behaviors.	Surveys; incident reports; medication logs	Residents (at baseline *n* = 34; follow up *n* = 29), staff (*n* = 16), and family (*n* = 15)	Age 74–92, male veterans, level of dementia not specified	MFP QoL	4
Detweiler et al. (2009); USA	To explore the effect of a dementia wander garden on scheduled psychiatric medications, changes in fall frequency and severity.	Medication logs; incident reports	Residents (at baseline and follow up *n* = 28)	Age 74–92, male veterans, level of dementia not specified	BF	4
Ford Murphy et al. (2010); USA	To reevaluate the findings of Detweiler et al. (2008) in more depth and to investigate whether there is a differential effect based on ambulation	Survey; number of garden visits; ambulatory condition	Residents (baseline *n* = 34; follow up *n* = 29)	Age 74–92, male veterans, level of dementia not specified	MFP	5
Qualitative studies						
De Boer et al. (2019); The Netherlands	From the perspective of the informal caregivers of people with dementia, exploring the positive and negative experiences with different types of NHs.	Interviews	Informal caregivers of residents (*n* = 43)	Greencare farm: age not specified, 23 women and 11 men, moderate dementia Traditional NH: age not specified, 18 women and 11 men, moderate dementia Regular small-scale living facility: 45 women and seven men, moderate dementia	MFP	5
Dreyfus et al. (2018); Australia	To study staff and family attitudes towards fences as a means of detaining people with dementia in residential aged care settings.	Focus groups	Managers (*n* = 12), nurse unit managers (*n* = 7), direct care workers (*n* = 6), and family members (*n* = 6)	Age, gender, and level of dementia of residents not specified	BF MFP QoL SSP	5
Evans et al. (2018); UK	To determine how care home managers negotiate the conflict between maintaining a safe environment while enabling the autonomy of residents with dementia.	Interviews	Care home managers (*n* = 18)	Age, gender, and level of dementia of residents not specified	MFP QoL	5
Fisher et al., (2018); UK	To investigate the impact that building design has upon the quality of life for residents of a care home who have dementia.	Interviews	Residents (*n* = 10) and staff (*n* = 5)	Age, gender, and level of dementia of residents not specified	MFP	4
Heggestad et al., (2013); Norway	To investigate how life in NHs may affect experiences of dignity among persons with dementia.	Observation; fieldnotes; interviews	Residents (*n* = 5 for interviews; *n* = 15 for observations)	Small-scale NH: age 79–99 years, gender not specified, mild to severe dementia Large-scale NH: age 73–92 years, gender not specified, level of dementia not specified	MFP QoL	5
Van Hecke et al. (2018); Belgium	To gain insight into how a dementia special care unit is used and experienced by its residents and what design aspects are important therein.	Observation; interviews	Residents (*n* = 4), staff (*n* = 4), director (*n* = 1), and architect (*n* = 1)	Age 86–94 years, all female, different levels of dementia	MFP SSP	5
Niemeijer et al., (2014); The Netherlands	To explore how clients in residential care experience surveillance technology and how it might influence autonomy.	Observation; conversations; interviews	Residents (*n* = 43), representative of clients (*n* = 1), and staff (*n* = 6)	Age, gender, and level of dementia of residents not specified	BF MFP SSP	5
Steele et al. (2020); Australia	To deepen human rights scholars’ and practitioners’ understanding of the drivers and facilitators of confinement in care homes.	Interviews; focus groups	Residents (*n* = 5), care partners (*n* = 19), care home professionals (*n* = 12), and lawyers and advocates (*n* = 9)	Age, gender, and level of dementia of residents not specified	MFP QoL SSP	5
Tufford et al. (2018); USA	To explore broader assumptions, connotations, and possibilities of built environments by comparing and contrasting the use of and philosophies regarding the locking of doors to those that have open doors.	Interviews; field observations	Management, health providers, support staff, informal care providers, union representatives, residents, and family members (*n* = 285)	Age, gender, and level of dementia of residents not specified	QoL	4
Wigg (2010); USA	To examine the impact of locked door environments on residents with dementia and compare their experiences with residents with dementia living in an unlocked environment.	Observations; interviews; field notes; video data	Residents (*n* = 60)	Closed NH: age between late 60s into 90s, eight men, 22 women, middle to late-stage dementia Open NH: age 63–95; varying from 15 to 10 men and 15 to 20 women, level of dementia not specified	BF MFP SSP	5
Mixed methods study						
Liao et al. (2018); USA	To evaluate the effects of garden visits on mood, social interaction, cognition, and behavioral problems and determine what type of behavioral problems and cognitive abilities among people with dementia may improve after visiting a garden.	Surveys; open-end questions	Staff (*n* = 42)	Age and gender not specified, mild and/or moderate and/or severe dementia.	BF MFP DF	5

*Notes*: MMAT = Mixed Methods Appraisal Tool; NH = nursing home; BF = bodily functions; MFP = mental functions and perception; ED = existential dimension; QoL = quality of life; SSP = social and societal participation; DF = daily functioning.

^a^MMAT scores could range from 0 (very poor quality) to 5 (excellent quality).

**Figure 2.**
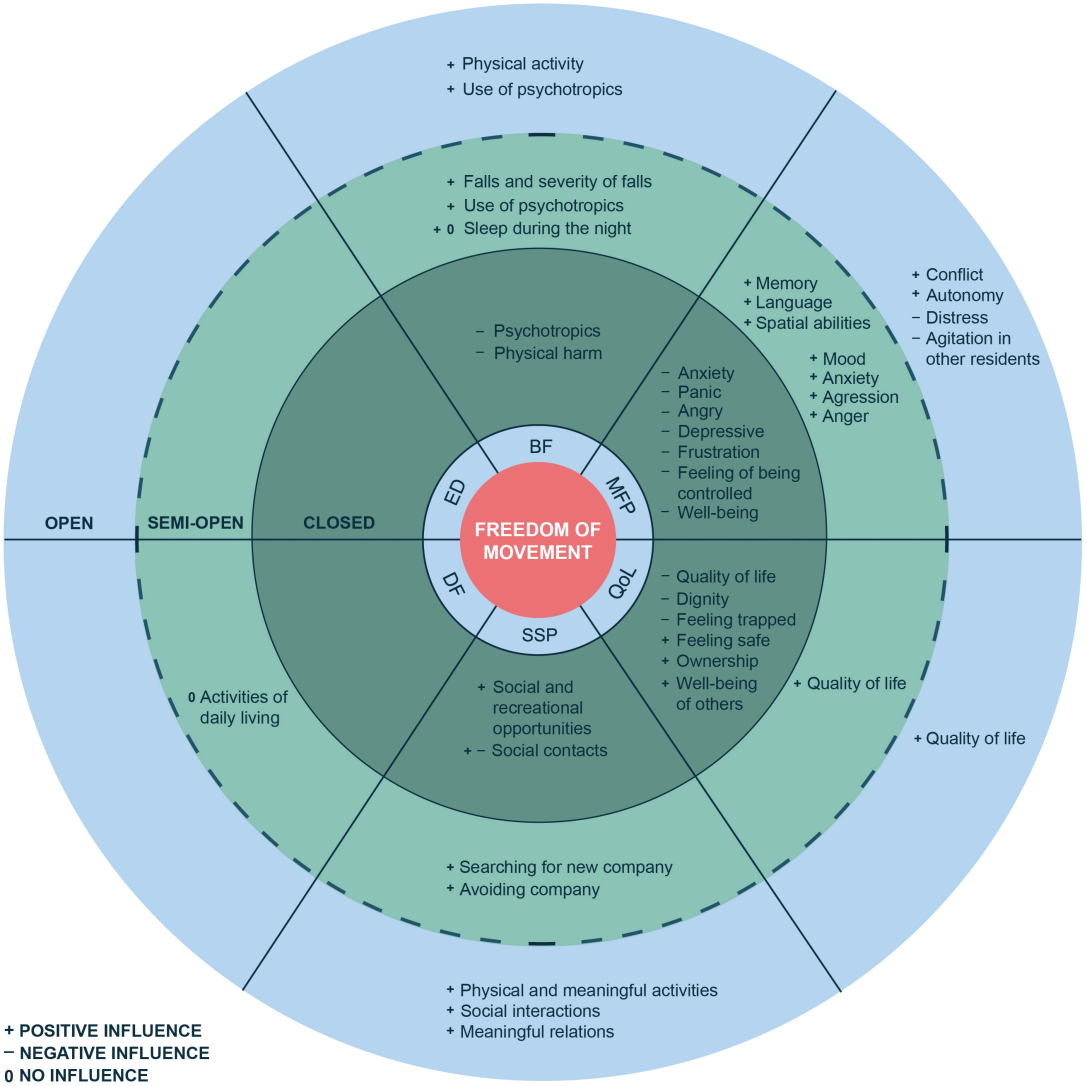
Overview of findings per level of freedom of movement (open, semiopen, and closed settings), categorized by the six dimensions of Positive Health.*Note:* BF = bodily functions; MFP = mental functions and perception; ED = existential dimension; QoL = quality of life; SSP = social and societal participation; DF = daily functioning.

An overview of the findings, categorized by the six dimensions of Positive Health and the three levels of freedom of movement, is shown in Figure 2. Overall, compared to closed NH settings, freedom of movement in semiopen and open settings may have a positive influence on people with dementia on four health dimensions as described by Huber et al. (2016): bodily functions, mental functions and perception, quality of life, and social and societal participation. In particular, increase in freedom of movement is related to a decrease in the use of psychotropic medication and the number and severity of falls. Furthermore, an increase in quality of life, social contact, some aspects of cognitive functioning such as long-term memory, language, and autonomy is found among NH residents with dementia living in semiopen and open settings compared to a closed setting. The influence of freedom of movement on daily functioning and the existential dimension remains unclear.

### Bodily Functions

Seven articles studied the influence of freedom of movement on the bodily functions of people with dementia living in NHs (Table 2). Findings of a Dutch study revealed that residents in green care farms (open setting) were more regularly outdoors and more physically active than residents in traditional NHs (both semiopen and closed settings; De Boer, Hamers, Zwakhalen, Tan, Beerens et al., 2017). Adding a free-access enclosed garden to a closed setting had the effect of reducing the number and severity of falls (Detweiler et al., 2009), as well as the use of psychotropics, such as antipsychotics and antidepressants (Detweiler et al., 2009; Wigg, 2010). On the contrary, in a closed setting, residents were sedated because of their frustrations surrounding their limited freedom of movement because of fences (Dreyfus et al., 2018). Fences also caused physical harm, as residents tried to climb over them to escape their confinement (Dreyfus et al., 2018). The findings regarding sleep are mixed; studies showed both no influence (Liao et al., 2018) or a positive influence of increasing freedom of movement on reduced restlessness during the night (Niemeijer et al., 2014).

### Mental Functions and Perception

Eleven studies reported an influence of freedom of movement on mental functions and perception (Table 2). Giving residents free access to an enclosed garden within a closed setting, improved long-term memory, language abilities, spatial abilities, and mood (Liao et al., 2018). Also, mean agitation levels decreased as well as levels of aggression, anger, and anxiety (Detweiler et al., 2008; Evans et al., 2018; Liao et al., 2018). Median agitation levels increased when the garden was not as accessible as usual (Ford Murphy et al., 2010). In open settings, residents can (choose to) go outside independently, which has shown to avoid the potential for conflict and anxiety inherent in trying to open a locked door (Wigg, 2010). Informal caregivers were positive about the autonomy of residents living in open settings (De Boer et al., 2019). Residents in a closed setting often panicked or became angry and verbally expressed their frustrations towards locked doors, showed depressive behaviors, or withdrew (Wigg, 2010). Residents also expressed feelings of being controlled and the need to ask permission to go outside (Fisher et al., 2018). Residents want to get out, which makes their behavior worse because they feel trapped; “this entrapment causes frustration, frustration causes anxiety, and anxiety accelerates everything” (Dreyfus et al., 2018). Programs such as walking activities were not sufficient to sustain mental and emotional well-being and did not compensate for the distress caused by being locked up (Dreyfus et al., 2018). Similarly, windows to outside spaces caused frustration in some residents living in a closed setting because everything happening behind these windows is visible, yet unreachable for them (Van Hecke et al., 2018). On the contrary, one study found that increased distress as a result of getting lost when given more freedom of movement or could trigger agitation in other residents who are not allowed to go outside a closed setting (Niemeijer et al., 2014).

### Quality of Life

Seven articles addressed the influence of freedom of movement on quality of life (Table 2). De Boer, Hamers, Zwakhalen, Tan et al. (2017) found that residents in green care farms (open settings) had a higher quality of life score compared with residents of traditional NHs (semiopen and closed settings). Staff and family members also stated that the resident’s quality of life improved after opening an enclosed wander garden (semiopen setting) compared with before (closed setting; Detweiler et al., 2008). Although NHs were aware that locked doors affect the quality of life of residents, increased risk perception of managers could result in an overuse of locks which hinder the quality of life (Tufford et al., 2018). Residents in a closed setting expressed a feeling of captivity related to a loss of dignity and quality of life (Heggestad et al., 2013; Steele et al., 2020). Finally, Dreyfus et al. (2018) found that many family members did not like fences used to keep residents in, feeling that their relative was a prisoner. On the contrary, according to some managers, fences can also have a positive effect on quality of life; they can help anxious residents feel safe and secure and provide a sense of ownership over a space and protecting them from unwanted outsiders wandering in (Dreyfus et al., 2018).

### Social and Societal Participation

Seven articles mentioned social and societal participation as an outcome related to freedom of movement (Table 2). Residents with dementia in open settings were observed to participate more in physical and meaningful activities and social interactions than residents in semiopen and closed settings (De Boer, Hamers, Zwakhalen, Tan, Beerens et al., 2017; De Boer, Hamers, Zwakhalen, Tan et al., 2017). Residents in closed settings were denied access to the full range of social and recreational opportunities (Steele et al., 2020), whereas some residents would like to have more social contact with residents outside to avoid the social dynamics within the closed setting or to have different kinds of conversations (Van Hecke et al., 2018). When freedom of movement was increased, a study found that NH residents with dementia were more able to retreat to new spaces to avoid the company of coresidents or to search for new company (Niemeijer et al., 2014). An open setting could also stimulate the relationship between residents and care providers (Wigg, 2010). From the perspective of staff and family, fences that can be seen through allow residents to socialize with the outside world and facilitate contact with people passing by (Dreyfus et al., 2018).

### Daily Functioning

One study examined the influence of freedom of movement on the aspect ADL among a group of NH residents with dementia who had free access to a garden (semiopen setting) compared to a group of residents who did not have free access to a garden (closed setting; Liao et al., 2018). ADL in this study was measured with one single item without ADL having been further specified. No differences between the two groups were observed.

### Existential Health

None of the included articles studied the influence of freedom of movement on existential health.

## Discussion and Implications

This systematic review aimed to investigate what is known in the current scientific literature about the influence of freedom of movement on all health dimensions among people with dementia living in NHs. Overall, results indicate that, compared to closed NH settings, freedom of movement in semiopen and open settings may have a positive influence on bodily functions, mental functions and perception, quality of life, and social and societal participation. The influence of freedom of movement on daily functioning and on the existential dimension remains unclear.

In addition to freedom of movement, seven of the included studies observed factors related to a garden that may have a positive influence on health (De Boer, Hamers, Zwakhalen, Tan, Beerens et al., 2017; De Boer, Hamers, Zwakhalen, Tan et al., 2017; De Boer et al., 2019; Detweiler et al., 2008; Detweiler et al., 2009, Ford Murphy et al., 2010; Liao et al., 2018). For example, another literature study showed that, apart from freedom of movement, a garden characterized by a green environment and being outside can have a positive effect on the health of NH residents with dementia (Van den Berg et al., 2020). Indeed, according to Liao et al. (2018), staff stated that the positive influence on health in residents with dementia occurred through garden visits, caused by the multisensory stimuli of the natural environment, which was also found in the study of Dahlkvist et al. (2020). However, in all of these studies, freedom of movement was a substantial factor associated with the found health outcomes. In the studies of Detweiler et al. (2008), Detweiler et al. (2009), and Ford Murphy et al. (2010), an explicit choice was made for an unlocked garden because having continued access to an unlocked door into the garden may bring about repeated temporary reductions in agitation. Liao et al. (2018), derived the health outcomes from comparisons between a free garden use group (with no closed doors to the garden) and an unfree garden use group (with closed doors to the garden). In the studies of De Boer, Hamers, Zwakhalen, Tan, Beerens et al. (2017), De Boer, Hamers, Zwakhalen, Tan et al. (2017), De Boer et al., (2019), different levels of freedom of movement were part of the three settings examined. According to De Boer, freedom of movement could not be identified as the only factor that explained the positive effect on health, because the open setting provided an environment where residents had the opportunity to participate in outdoor, domestic, and other activities integrated into everyday life. In conclusion, it is likely that various factors have collectively contributed to the positive impact of freedom of movement on health.

In the included articles, the perspectives of NH residents with dementia themselves and the perspectives of those involved with these residents, such as family members, staff, or managers, were, overall, similar regarding the positive influence of increased freedom of movement on health. However, according to the perspective of managers, restriction of freedom of movement could also have a positive impact on the health of NH residents or the well-being of others. This was mentioned by Dreyfus (2018), where managers argued that restriction of freedom of movement by fences could help anxious residents feel safe and secure and protect the general population from the risks posed by residents with dementia wandering off NH grounds. Another included study found that managers prioritized the safety of residents through locked wards above increased freedom of movement for residents, although they recognized the negative consequence of locked doors on autonomy for these residents (Evans et al., 2018). These perspectives of managers may hinder increasing freedom of movement and conflict with personal choice and autonomy of residents as part of person-centered care (Davies et al., 2022; Fazio et al., 2018).

### Strengths and Limitations

This is the first study that systematically combined the scientific literature about the influence of freedom of movement on all dimensions of the health of NH residents with dementia. To date, no such complete overview exists. We systematically searched five scientific databases and assessed all 16 included studies on their methodological quality. These studies were conducted in several countries around the world and may therefore differ in terms of NH characteristics, values, regulations, and staffing. Such factors may have influenced study results. On the other hand, because all these NHs meet the international definition of a NH, this review provides a broad picture of freedom of movement for people with dementia in NHs. When interpreting the findings, however, some limitations must be taken into account. First, findings from five studies may not be completely generalizable to the current NH population with dementia. Three studies included only male veteran residents (Detweiler et al., 2008, 2009; Ford Murphy et al., 2010), whereas the majority of the NH population consists of women. In two other studies, a matching procedure was used to increase comparability between residents in different types of settings in terms of the level of dementia (De Boer, Hamers, Zwakhalen, Tan, Beerens et al., 2017; De Boer, Hamers, Zwakhalen, Tan et al., 2017). The consequence of this matching procedure may imply that the findings are not generalizable to NH residents with moderately severe or severe dementia. Additionally, we noted that in the included articles, little attention is paid to other differences in characteristics of NH residents, including physical ability to move and type of dementia related to freedom of movement and the impact on health. For example, dementia is a disease that covers a wide range of medical conditions, with the most common forms being Alzheimer’s disease, vascular dementia, dementia of Lewy bodies, and frontal–temporal lobe dementia (Hobson, 2019). Each form has its own specific symptoms, which can influence the potential impact of freedom of movement on health.

### Implications for Future Research and Practice

With the increasing demand for high-quality and safe long-term care for people with dementia, it is time to challenge the assumption that it is necessary, safe, and healthy to keep people with dementia in a locked environment. In line with these developments, the findings of this review are a valuable addition to the state-of-the-art scientific evidence on health benefits of freedom of movement. It is recommended that future studies assess freedom of movement in a prospective manner to uncover mechanisms by which freedom of movement may contribute to health. Moreover, future studies should consider the influence on health of varying levels of freedom. Such research should also consider the diversity of people with dementia living in NHs that may impact (the decision to) independent movement from one place to another, such as the type and severity of dementia and physical abilities to move.

## Conclusions

To date, the scientific literature suggests freedom of movement in semiopen and open settings, compared to closed settings, may have a positive influence on the health of NH residents with dementia, in particular on bodily functions, mental functions and perception, quality of life, and social and societal participation. In several of the included studies, however, freedom of movement was part of a larger context studied in which other factors also may have resulted in health benefits. For this reason, more research is needed in order to unravel the underlying mechanisms of the positive influence of freedom of movement on health.

## Supplementary Material

gnac114_suppl_Supplementary_Material
