# Supplementary material for: Influence of Freedom of Movement on the Health of People With Dementia: A Systematic Review
Source: Gerontologist. 2022 Aug 5;63(8):1351–64. doi: 10.1093/geront/gnac114 (PMC10474594; doi:10.1093/geront/gnac114)
Supplement: gnac114_suppl_Supplementary_Material [file gnac114_suppl_supplementary_material.docx]

**ONLINE SUPPLEMENTARY MATERIAL**

**Search string**

| Embase.com | |
| --- | --- |
| ('dementia'/exp OR 'aged'/de OR 'geriatrics'/exp OR 'institutionalized elderly'/de OR 'home for the aged'/de OR 'nursing home'/de OR 'nursing home patient'/de OR 'elderly care'/exp OR senescence/de OR (dement* OR alzheimer* OR elderly OR psychogeriatr* OR geropsychiatr* OR geriatr* OR ((home* OR housing) NEAR/3 (aged OR senior*)) OR nursing home* OR ((old* OR aged) NEXT/1 (adult* OR people OR age OR women OR men OR care)) OR geronto* OR senescence OR aging):ab,ti) AND (('movement (physiology)'/de AND freedom/de) OR 'physical restraint'/de OR 'physical mobility'/de OR gardening/de OR 'global positioning system'/de OR (((freedom*) NEAR/3 (movement* OR depriv* OR restrict* OR restrain*)) OR (movement* NEAR/3 depriv*) OR walkabout OR walk about OR getting lost OR outside world OR ((unlock* OR lock* OR closed OR open*) NEAR/3 doors) OR ((physical* OR mechanical*) NEAR/3 (Constraint* OR restrain* OR mobilit* OR immobilit*)) OR restraint free OR constraint free OR ((electronic* OR technolog* OR gps) NEAR/3 surveillan*) OR seclusion OR enclos* OR outdoor* OR garden* OR going out* OR wandering around OR walking around OR global positioning system):ab,ti OR (restraint* OR constraint*):ti) AND ('residential home'/de OR 'residential care'/de OR 'nursing home'/de OR 'nursing home patient'/de OR 'nursing home personnel'/de OR 'long term care'/de OR 'institutionalization'/de OR 'institutionalized person'/de OR 'institutionalized elderly'/de OR 'institutional care'/de OR 'home for the aged'/de OR 'assisted living facility'/de OR 'ward'/de OR 'gerontopsychiatry'/de OR (((resident* OR long term OR geriatr*) NEXT/1 (home* OR facilit* OR care OR housing OR setting* OR aged care)) OR nursing home* OR nursing facilit* OR inpatient* OR (care unit* NOT Intensive care unit*) OR care home* OR institutional* OR ((home* OR housing) NEAR/3 (aged OR senior*)) OR assisted living OR ward OR wards OR gerontopsychiatr* OR geropsychiatr* OR gerontopsychiatr* OR psychogeriatr* OR psychogeriatr* OR dementia care):ab,ti) NOT ([Conference Abstract]/lim AND [1800 2017]/py) AND [English]/lim | |
| Medline ALL Ovid | |
| (exp Dementia/ OR exp Aged/ OR Geriatric Psychiatry/ OR Geriatrics/ OR Homes for the Aged/ OR exp Nursing Homes/ OR Geriatric Nursing/ OR Aging/ OR (dement* OR alzheimer* OR elderly OR psychogeriatr* OR geropsychiatr* OR geriatr* OR ((home* OR housing) ADJ3 (aged OR senior*)) OR nursing home* OR ((old* OR aged) ADJ (adult* OR people OR age OR women OR men OR care)) OR geronto* OR senescence OR aging).ab,ti.) AND ((Movement/ AND Freedom/) OR Restraint, Physical/ OR Gardening/ OR Geographic Information Systems/ OR (((freedom*) ADJ3 (movement* OR depriv* OR restrict* OR restrain*)) OR (movement* ADJ3 depriv*) OR walkabout OR walk about OR getting lost OR outside world OR ((unlock* OR lock* OR closed OR open*) ADJ3 doors) OR ((physical* OR mechanical*) ADJ3 (Constraint* OR restrain* OR mobilit* OR immobilit*)) OR restraint free OR constraint free OR ((electronic* OR technolog* OR gps) ADJ3 surveillan*) OR seclusion OR enclos* OR outdoor* OR garden* OR going out* OR wandering around OR walking around OR global positioning system).ab,ti. OR (restraint* OR constraint*).ti.) AND (Residential Facilities/ OR Nursing Homes/ OR Long Term Care/ OR Institutionalization/ OR Homes for the Aged/ OR Assisted Living Facilities/ OR Geriatric Psychiatry/ OR (((resident* OR long term OR geriatr*) ADJ (home* OR facilit* OR care OR housing OR setting* OR aged care)) OR nursing home* OR nursing facilit* OR inpatient* OR (care unit* NOT Intensive care unit*) OR care home* OR institutional* OR ((home* OR housing) ADJ3 (aged OR senior*)) OR assisted living OR ward OR wards OR gerontopsychiatr* OR geropsychiatr* OR gerontopsychiatr* OR psychogeriatr* OR psychogeriatr* OR dementia care).ab,ti.) AND english.la. | |
| PsycINFO Ovid | |
| (exp Dementia/ OR 300.ag. OR Geriatric Psychiatry/ OR Geriatrics/ OR exp Nursing Homes/ OR Elder Care/ OR Aging/ OR (dement* OR alzheimer* OR elderly OR psychogeriatr* OR geropsychiatr* OR geriatr* OR ((home* OR housing) ADJ3 (aged OR senior*)) OR nursing home* OR ((old* OR aged) ADJ (adult* OR people OR age OR women OR men OR care)) OR geronto* OR senescence OR aging).ab,ti.) AND (Physical Restraint/ OR (((freedom*) ADJ3 (movement* OR depriv* OR restrict* OR restrain*)) OR (movement* ADJ3 depriv*) OR walkabout OR walk about OR getting lost OR outside world OR ((unlock* OR lock* OR closed OR open*) ADJ3 doors) OR ((physical* OR mechanical*) ADJ3 (Constraint* OR restrain* OR mobilit* OR immobilit*)) OR restraint free OR constraint free OR ((electronic* OR technolog* OR gps) ADJ3 surveillan*) OR seclusion OR enclos* OR outdoor* OR garden* OR going out* OR wandering around OR walking around OR global positioning system).ab,ti. OR (restraint* OR constraint*).ti.) AND (Residential Care Institutions/ OR Nursing Homes/ OR Long Term Care/ OR Institutionalization/ OR Assisted Living/ OR Geriatric Psychiatry/ OR (((resident* OR long term OR geriatr*) ADJ (home* OR facilit* OR care OR housing OR setting* OR aged care)) OR nursing home* OR nursing facilit* OR inpatient* OR (care unit* NOT Intensive care unit*) OR care home* OR institutional* OR ((home* OR housing) ADJ3 (aged OR senior*)) OR assisted living OR ward OR wards OR gerontopsychiatr* OR geropsychiatr* OR gerontopsychiatr* OR psychogeriatr* OR psychogeriatr* OR dementia care).ab,ti.) AND english.la. | |
| Web of science Core Collection |  |
| AB=(((dement* OR alzheimer* OR elderly OR psychogeriatr* OR geropsychiatr* OR geriatr* OR ((home* OR housing) NEAR/2 (aged OR senior*)) OR nursing home* OR ((old* OR aged) NEAR/1 (adult* OR people OR age OR women OR men OR care)) OR geronto* OR senescence OR aging)) AND ((((freedom*) NEAR/2 (movement* OR depriv* OR restrict* OR restrain*)) OR (movement* NEAR/2 depriv*) OR walkabout OR walk about OR getting lost OR outside world OR ((unlock* OR lock* OR closed OR open*) NEAR/2 doors) OR ((physical* OR mechanical*) NEAR/2 (Constraint* OR restrain* OR mobilit* OR immobilit*)) OR restraint free OR constraint free OR ((electronic* OR technolog* OR gps) NEAR/2 surveillan*) OR seclusion OR enclos* OR outdoor* OR garden* OR going out* OR wandering around OR walking around OR global positioning system) OR (restraint* OR constraint*):ti) AND ((((resident* OR long term OR geriatr*) NEAR/1 (home* OR facilit* OR care OR housing OR setting* OR aged care)) OR nursing home* OR nursing facilit* OR inpatient* OR (care unit* NOT Intensive care unit*) OR care home* OR institutional* OR ((home* OR housing) NEAR/2 (aged OR senior*)) OR assisted living OR ward OR wards OR gerontopsychiatr* OR geropsychiatr* OR geronto psychiatr* OR psychogeriatr* OR psycho geriatr* OR dementia care))) |  |
| Cochrane CENTRAL register of trials |  |
| ((dement* OR alzheimer* OR elderly OR psychogeriatr* OR geropsychiatr* OR geriatr* OR ((home* OR housing) NEAR/3 (aged OR senior*)) OR nursing home* OR ((old* OR aged) NEXT/1 (adult* OR people OR age OR women OR men OR care)) OR geronto* OR senescence OR aging):ab,ti) AND ((((freedom*) NEAR/3 (movement* OR depriv* OR restrict* OR restrain*)) OR (movement* NEAR/3 depriv*) OR walkabout OR walk about OR getting lost OR outside world OR ((unlock* OR lock* OR closed OR open*) NEAR/3 doors) OR ((physical* OR mechanical*) NEAR/3 (Constraint* OR restrain* OR mobilit* OR immobilit*)) OR restraint free OR constraint free OR ((electronic* OR technolog* OR gps) NEAR/3 surveillan*) OR seclusion OR enclos* OR outdoor* OR garden* OR going out* OR wandering around OR walking around OR global positioning system):ab,ti OR (restraint* OR constraint*):ti) AND ((((resident* OR long term OR geriatr*) NEXT/1 (home* OR facilit* OR care OR housing OR setting* OR aged care)) OR nursing home* OR nursing facilit* OR inpatient* OR (care unit* NOT Intensive care unit*) OR care home* OR institutional* OR ((home* OR housing) NEAR/3 (aged OR senior*)) OR assisted living OR ward OR wards OR gerontopsychiatr* OR geropsychiatr* OR geronto psychiatr* OR psychogeriatr* OR psychogeriatr* OR dementia care):ab,ti) |  |
